# Identifying Increases in Activity of the Human RVLM Through MSNA-Coupled fMRI

**DOI:** 10.3389/fnins.2019.01369

**Published:** 2020-01-21

**Authors:** Vaughan G. Macefield, Luke A. Henderson

**Affiliations:** ^1^Human Autonomic Neurophysiology Laboratory, School of Medicine, Baker Heart and Diabetes Institute, Melbourne, VIC, Australia; ^2^Department of Physiology, School of Biomedical Sciences, The University of Melbourne, Melbourne, VIC, Australia; ^3^Discipline of Anatomy and Histology, School of Medical Sciences, The University of Sydney, Sydney, NSW, Australia; ^4^Brain and Mind Centre, The University of Sydney, Sydney, NSW, Australia

**Keywords:** muscle sympathetic nerve activity, functional magnetic resonance imaging, rostral ventrolateral medulla, blood pressure, human

## Abstract

**Aim:**

We initially developed concurrent recording of muscle sympathetic nerve activity (MSNA) and functional magnetic resonance imaging (fMRI) of the brain to functionally identify the human homolog of the rostral ventrolateral medulla (RVLM). Here we summarize the cortical and subcortical connections to the RVLM, as identified using MSNA-coupled fMRI.

**Methods:**

MSNA was recorded via tungsten microelectrodes inserted into the peroneal nerve. Gradient echo, echo-planar fMRI was performed at 3T (Philips Achieva). 200 volumes (46 axial slices (TR = 8 s, TE = 4 s, flip angle = 90°, raw voxel size = 1.5 × 1.5 × 2.75 mm) were collected in a 4 s-ON, 4 s-OFF sparse sampling protocol and MSNA measured in each 1 s epoch in the 4-s period between scans. Blood oxygen level dependent (BOLD) signal intensity was measured in the corresponding 1 s epoch 4 s later to account for peripheral neural conduction and central neurovascular coupling delays.

**Results:**

BOLD signal intensity was positively related to bursts of MSNA in the RVLM, dorsomedial hypothalamus (DMH), ventromedial hypothalamus (VMH), insula, dorsolateral prefrontal cortex (dlPFC), posterior cingulate cortex (PCC), and precuneus, and negatively related in the caudal ventrolateral medulla (CVLM), nucleus tractus solitarius (NTS), and the midbrain periaqueductal gray (PAG). During physiological increases in MSNA (tonic muscle pain), MSNA-coupled BOLD signal intensity was greater in RVLM, NTS, PAG, DMH, dlPFC, medial prefrontal cortex (mPFC), precuneus, and anterior cingulate cortex (ACC) than at rest. During pathophysiological increases in MSNA [obstructive sleep apnoea (OSA)] signal intensity was also higher in dlPFC, mPFC, ACC, and precuneus than in controls. Conversely, signal intensity was lower in RVLM in OSA than in controls, which we interpret as reflecting a withdrawal of active inhibition of the RVLM.

**Conclusion:**

These results suggest that multiple cortical and subcortical areas are functionally coupled to the RVLM, which in turn is functionally coupled to the generation of spontaneous bursts of MSNA and their augmentation during physiological and pathophysiological increase in vasoconstrictor drive.

## The Role of the RVLM in the Regulation of Blood Pressure

From classic studies of decerebrate animals, it has long been known that the control of blood pressure requires an intact brainstem, in particular the medulla oblongata; section of the spinal cord immediately below the medulla leads to a precipitous fall in blood pressure, while section of the brainstem at the pontomedullary junction does not. The maintenance of blood pressure within a relatively narrow range depends on the integrity of a simple reflex arc, the baroreflex. Baroreceptors – mechanoreceptors located within the carotid sinus and aortic arch that are sensitive to radial distension of the arterial wall and hence to intraluminal pressure – detect the pulsatile fluctuations in blood pressure and, via the glossopharyngeal and vagus nerves, send excitatory synaptic projections to the primary visceral sensory nucleus, the nucleus of the solitary tract (NTS). Second-order neurones within NTS then send excitatory projections to neurones of the caudal ventrolateral medulla (CVLM), which exerts tonic inhibitory control of the rostral ventrolateral medulla (RVLM) (Dampney et al., 2003a, b; Guyenet, 2006). It is well known that the RVLM plays a critical role in maintaining absolute levels of blood pressure, as well as being essential for the beat-to-beat control of blood pressure: almost all sympathetic vasoconstrictor neurones originate in the RVLM and electrolytic lesions of the RVLM result in precipitous falls in blood pressure (Kumada et al., 1979; Dampney and Moon, 1980). However, there is evidence suggesting that this view of the RVLM being critical to the maintenance of blood pressure is incorrect. Recently, Wenker et al. (2017) showed that laser-induced inhibition of archaerhodopsin-expressing RVLM neurones failed to drop blood pressure significantly in conscious rats. However, the authors do acknowledge that just over 50% of the neurones expressed archaerhodopsin, so it is possible that inadequate inhibition was produced during laser stimulation.

Given that the RVLM is the primary (albeit not exclusive) output nucleus for sympathetic vasoconstrictor drive to the muscle, splanchnic, and renal vascular beds (Dampney and McAllen, 1988; McAllen et al., 1995), and thereby plays an important role in the ongoing regulation of total peripheral resistance and blood pressure, measuring sympathetic vasoconstrictor nerve activity in the periphery can be used to infer the state of activity of the RVLM, as well as other nuclei with spinally projecting neurones – such as the paraventricular nucleus of the hypothalamus (PVN), which sends direct projections to the spinal cord as well as to RVLM (Shafton et al., 1998; Pyner and Coote, 2000). However, given that spinally projecting PVN neurones do not respond to baroreceptor input in the conscious rabbit (Dampney et al., 2003b) it is likely that this nucleus contributes little to resting vasoconstrictor drive to vascular beds involved in regulating total peripheral resistance, such as those in skeletal muscle.

Sympathetic outflow to the muscle vascular bed can be recorded directly in humans via a tungsten microelectrode inserted percutaneously into an accessible peripheral nerve, an invasive technique known as microneurography. Muscle sympathetic nerve activity (MSNA) occurs as spontaneous bursts that show a strong temporal coupling to the heart beat; bursts occur in the intervals between heart beats, with longer cardiac intervals being associated with lower diastolic pressures and a greater incidence and amplitude of bursts of MSNA (Macefield, 2013). Although there is no association between resting MSNA and blood pressure in normotensive humans (Joyner et al., 2010), it is well established that elevated MSNA contributes to the development of neurogenic hypertension (Wallin et al., 1973; Grassi et al., 1998; Schlaich et al., 2004).

## Functional Identification of the Human RVLM Using MSNA-Coupled fMRI

Nearly 10 years ago we published our first paper (Macefield and Henderson, 2010) on MSNA-coupled functional magnetic resonance imaging (fMRI), in which we combined direct recordings of MSNA with fMRI of the brainstem. Our approach, in which fluctuations in the incidence and amplitude of spontaneous bursts of MSNA recorded in the periphery are used to identify covarying fluctuations in BOLD (blood oxygen level dependent) signal intensity, was used to identify individual nuclei in the brainstem responsible for the generation of the signal. In other words, recording the output signal at the same time as imaging the brain allowed us to identify the central source of the output signal. Given the technical difficulties of recording small nerve signals in a large magnetic field (3 Tesla, and we are now doing this at 7T), this had never before been attempted. Details of our methodology can be found elsewhere (Macefield and Henderson, 2010, 2016, 2019), but briefly spontaneous bursts of MSNA were recorded via a tungsten microelectrode inserted percutaneously into a muscle fascicle of the right common peroneal nerve in supine participants, and neural activity amplified, filtered (2 × 10^4^, 0.3–5.0 kHz; NeuroAmpEx, ADInstruments, Sydney, NSW, Australia) and sampled on computer at 10 kHz (PowerLab 16S and LabChart 7 software, ADInstruments). The head was enclosed in a 32-channel SENSE head coil and a sparse gradient-gradient echo sampling protocol was performed: 200 volumes (TR = 8 s, TE = 4 s, flip angle = 90°, raw voxel size = 1.5 × 1.5 × 2.75 mm) were acquired over 27 min, each volume comprising 46 axial slices collected in a caudal to rostral direction and extending from the top of the cervical spinal cord to the vertex. Each 8 s TR period was composed of an initial 4 s “ON” phase during which the entire fMRI volume was collected, followed by a non-scanning “OFF” phase of 4 s, in which bursts of MSNA were measured in each of the four 1-s epochs. BOLD signal intensity (SPM12, uncorrected *p* < 0.001) was measured in each of the four 1-s epochs in the subsequent 4-s period to account for the ∼1 s required for arrival of the sympathetic volley at the peripheral recording site (Fagius and Wallin, 1980) and the ∼5 s hemodynamic delay between the neuronal activity and generation of the BOLD signal (Logothetis et al., 2001). Importantly, we do not use a region-of-interest approach: rather, areas of the brain are identified as being involved in the regulation of MSNA because the spontaneous fluctuations in BOLD signal intensity covaried with the spontaneous fluctuations in burst amplitude of the MSNA signal. In other words, these areas “popped out” because of their temporal coupling to the bursts of MSNA.

**FIGURE 1:**
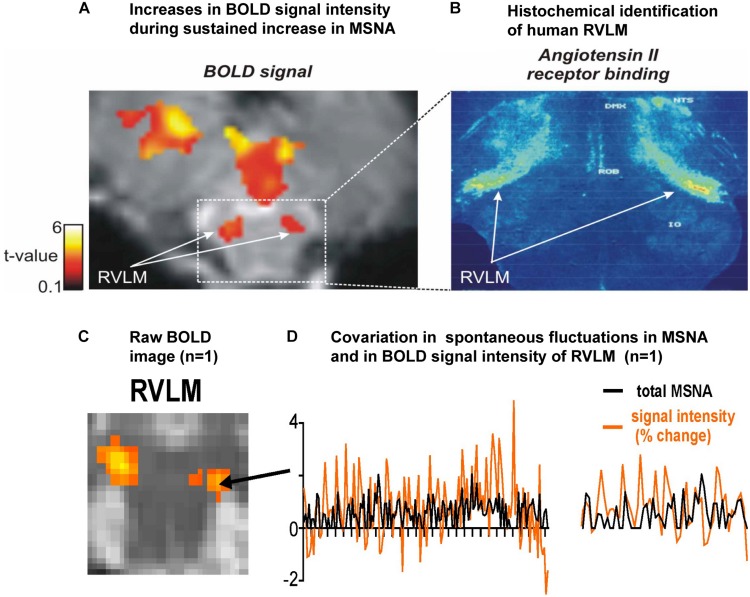
**(A)** Axial section of the rostral medulla showing bilateral increases in BOLD signal intensity in the dorsolateral medulla – the region of the human rostral ventrolateral medulla (RVLM) – during sustained increases in MSNA during a set of three maximal inspiratory breath-holds in 15 participants. **(B)** Histochemical identification of the human RVLM based on the high density of Angiotensin II receptor binding (data reproduced with permission from Allen et al., 1998). **(C)** Bilateral increases in MSNA-coupled BOLD signal intensity in the region of the RVLM. **(D)** Covariation in total MSNA, shown as the total amplitude of MSNA measured in each 1 s epoch, and BOLD signal intensity in the corresponding 1 s epochs, measured over 30 s in the region indicated in panel **(C)** for the same participant; an expanded section is shown on the right. Reproduced, with permission, from Macefield and Henderson (2019).

Figure 1A shows a bilateral increase in BOLD signal intensity in the medulla during three sets of maximal inspiratory breath holds – a manoeuver that causes a sustained increase in MSNA – in 15 participants (Macefield et al., 2006). We believe these clusters represent the human homolog of the RVLM: the human RVLM is not located in the ventrolateral part of the medulla, where it was first identified in the rabbit, but in the *dorsolateral* aspect of the medulla (Figure 1B). This is because the human RVLM, identified as such because of its high density of angiotensin II type IA receptors (AT1AR), is displaced by the large inferior olives in humans (Allen et al., 1998). In Figure 1C we show for one participant bilateral increases in MSNA-coupled BOLD signal intensity in these same areas: it can be seen that the BOLD signal and MSNA signal covary over time, shown for a 30 s sample for this same participant in Figure 1D.

**FIGURE 2:**
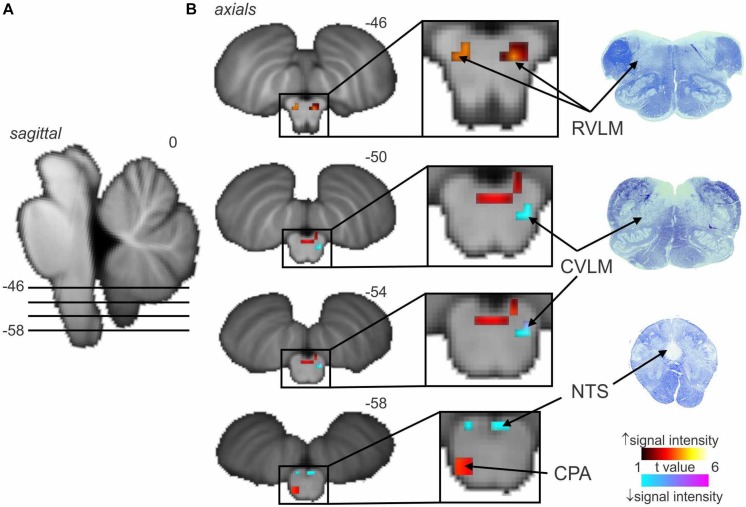
Significant increases (warm color scale) and decreases (cool color scale) in functional magnetic resonance imaging (fMRI) signal intensity within the brainstem correlated with spontaneous muscle sympathetic nerve activity (MSNA; total burst activity) in the resting state; data from seven experiments. **(A)** Significant changes are overlaid onto sagittal and axial slices of a T1-weighted brainstem template, with slice locations in Montreal Neurological Institute space indicated at the top right of each slice. **(B)** Equivalent histological sections. Note that spontaneous bursts of MSNA are associated with increases in signal intensity in the regions of the rostral ventrolateral medulla (RVLM) and caudal pressor area (CPA) and decreases in the region of the caudal ventrolateral medulla (CVLM) and nucleus tractus solitarius (NTS). Reproduced, with permission, from Macefield and Henderson (2019).

Figure 2 shows average data from eight participants. MSNA-coupled signal intensity was high in RVLM, yet low in the regions corresponding to NTS and CVLM. This makes sense, given that spontaneous bursts of MSNA only occur when diastolic blood pressure is low and hence the excitatory input to NTS from the arterial baroreceptors is also low. And, because NTS sends an excitatory projection to CVLM, BOLD signal intensity in this nucleus is also low. The opposite occurs when bursts of MSNA are absent when diastolic pressure is high. As such, these findings demonstrate the existence in humans of the serial NTS-CVLM-RVLM baroreflex circuit identified in experimental animals referred to above (Macefield and Henderson, 2010). It can also be seen that there is a unilateral (left) caudal medullary site in which BOLD signal intensity is high when bursts of MSNA occur: we suggest that this cluster corresponds to the caudal pressor area (CPA), an area known to send excitatory projections to RVLM (Dampney et al., 2003a, b).

**FIGURE 3:**
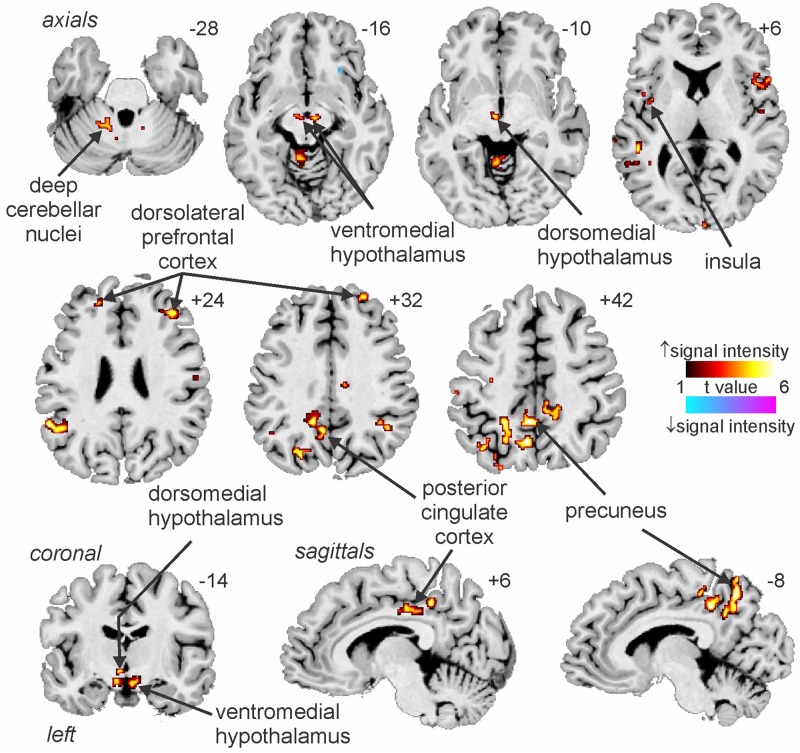
Increases and decreases in functional magnetic resonance imaging (fMRI) signal intensity correlated with muscle sympathetic nerve activity (MSNA) in 14 healthy participants. The hot color scale indicates regions in which signal intensity was high during periods of high MSNA and low during low MSNA. Conversely, the cool color scale indicates regions where signal intensity was high during low MSNA and low during high MSNA. Clusters are overlaid onto axial, coronal and sagittal slices of an individual subject’s T1-weighted anatomical and slice locations in Montreal Neurological Space are shown at the top right of each section. Reproduced, with permission, from James et al. (2013).

We have also used MSNA-coupled fMRI to identify structures above the brainstem. As shown in Figure 3, MSNA-coupled signal intensity was high in a number of discrete regions, including the left insula, left and right dorsolateral prefrontal cortex (dlPFC), posterior cingulate cortex (PCC), and precuneus. There was also significant MSNA-coupled signal intensity in the left dorsomedial hypothalamus (DMH) and both the left and right ventromedial hypothalamus (VMH). Neither DMH nor VMH send direct projections to the spinal cord, with DMH influencing sympathetic outflow via RVLM (DiMicco et al., 2002; Horiuchi et al., 2004; Wang et al., 2010) and VMH acting via DMH, midbrain periaqueductal gray (PAG), parabrachial nucleus, and NTS (ter Horst and Luiten, 1986; Canteras et al., 1994; Jansen et al., 1995). Note, however, that there was no signal in the PVN which – as noted above – is the one hypothalamic nucleus known to send direct projections to the spinal cord in parallel to those to RVLM (Shafton et al., 1998; Pyner and Coote, 2000).

**FIGURE 4:**
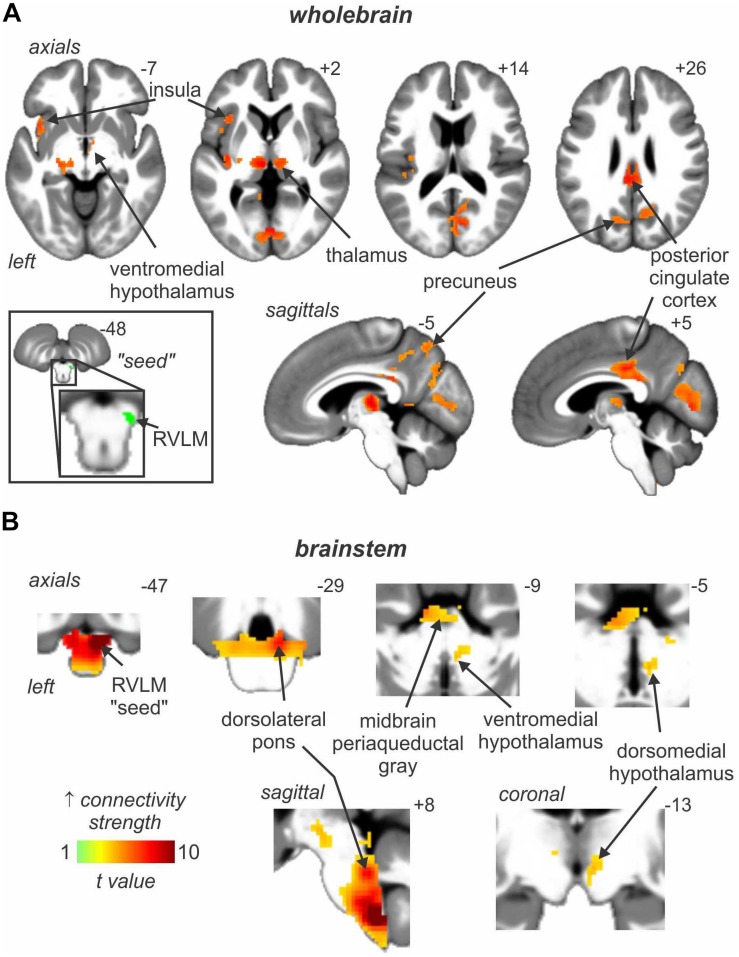
Brain regions in which signal intensity fluctuations at rest are significantly positively coupled to those in the rostral ventrolateral medulla (RVLM) in 29 healthy participants. Results from an analysis focused on rostral brain sites are shown in panel **(A)** whereas panel **(B)** focusses on the brainstem only. Significant positive signal couplings with the RVLM are indicated with the hot color scale and overlaid onto and axial, coronal and sagittal slices of a mean T1-weighted anatomical image. Slice locations in Montreal Neurological Space are shown at the top right of each section. Reproduced, with permission, from Macefield and Henderson (2019).

In addition to these areas being functionally coupled to bursts of MSNA, as shown in Figure 4 connectivity analysis revealed that the RVLM is functionally coupled to the anterior insula, PCC, precuneus, VMH and DMH, PAG, and dorsolateral pons (dlPons). This means that each of these areas are all functionally coupled to the generation of spontaneous bursts of MSNA and hence are likely to be involved in the generation of, and/or regulation of, MSNA at rest. We were surprised to find that changes in signal intensity in certain areas – including NTS, CVLM, CPA, DMH, and insula – were not symmetrical, although changes in RVLM, VMH, dlPFC, PCC, and precuneus were bilateral. We always recorded MSNA from the right common peroneal nerve, but given that both the incidence and amplitude of bursts of MSNA directed to the left and right legs are symmetrical, as demonstrated during bilateral recordings of MSNA (Sundlof and Wallin, 1977; El Sayed et al., 2012), any attempt to explain these side-to-side differences would be purely speculative. We direct the reader to our recent review in which we consider the functional significance of the sympathetic connectome we have identified (Macefield and Henderson, 2019).

## Changes in MSNA-Coupled Bold Signal Intensity During Physiological Increases in MSNA

We had already shown that BOLD signal intensity increases in RVLM during a maximal inspiratory breath-hold (see Figure 1A) while signal intensity in NTS and CVLM goes down (Macefield et al., 2006), and have shown that signal intensity increases in both NTS and RVLM during activation of metaboreceptors by inducing 6 min of post-exercise ischemia following 4 min of static handgrip exercise (Sander et al., 2010). Earlier studies had also shown that BOLD signal intensity increased in the medulla and dorsal pons during a Valsalva maneuver (Harper et al., 2000; Henderson et al., 2002), with increases in signal intensity in NTS and the parabrachial nucleus (to which NTS projects) being reported during a maximal inspiratory effort, isometric handgrip exercise and the Valsalva maneuver (Topolovec et al., 2004). However, in none of these studies had MSNA been recorded at the same time.

**FIGURE 5:**
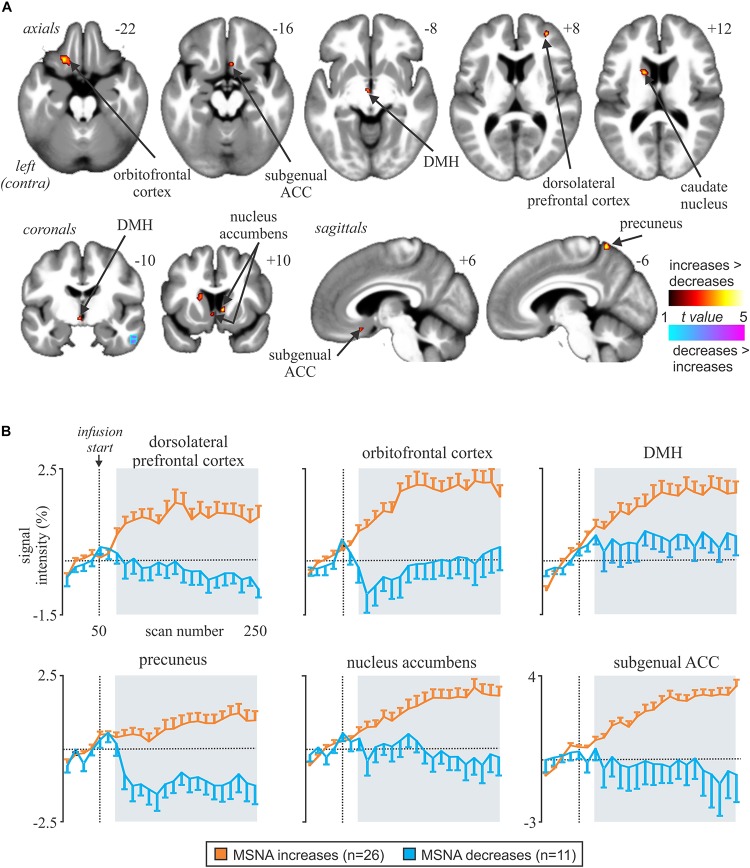
**(A)** Brain regions in which increases in BOLD signal intensity were greater or lower during tonic muscle pain. Significant clusters are overlaid onto a mean T1-weighted anatomical image set created from all 37 subjects. Slice locations in MNI space are indicated in the upper right of each image. The left side of the image is the side contralateral to the noxious stimulus. **(B)** Plots of mean (±SEM) percentage changes, measured every 5 min, at baseline and during pain (gray shading) in the increasing (orange) and decreasing (blue) groups. Reproduced, with permission, from Kobuch et al. (2017).

**FIGURE 6:**
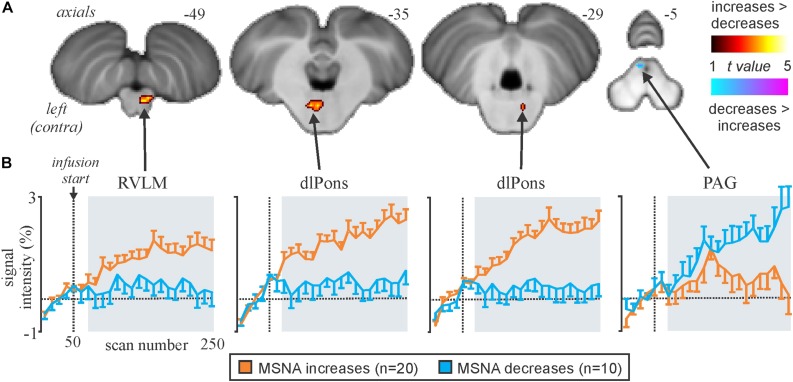
**(A)** Brainstem regions in which signal intensity increases were greater (hot color scale) or lower (cool color scale) in the increasing MSNA (*n*=20) compared with the decreasing MSNA (*n*=10) group during tonic muscle pain. Significant clusters were overlaid onto a mean SUIT T1-weighted anatomical image set created from 30 subjects. **(B)** Plots of mean (±SEM) percentage signal intensity changes during pain relative to the baseline period for significant clusters in the increasing MSNA (orange) and decreasing MSNA groups (blue). RVLM, rostroventrolateral medulla; dlPons, dorsolateral pons; PAG, midbrain periaqueductal gray. Reproduced, with permission, from Kobuch et al. (2017).

We recently examined functional changes in the brain during experimental muscle pain, induced by a 40-min infusion of hypertonic saline into a leg muscle, which causes a sustained increase in MSNA in some participants but a sustained decrease in others; the pattern is reproducible in a given individual and we recently showed that those in whom MSNA increased during tonic muscle pain exhibited increases in BOLD signal intensity in several areas (Kobuch et al., 2017, 2018), including the anterior insula and anterior medial prefrontal cortex (mPFC) on the left, and dlPFC and anterior cingulate cortex (ACC) on the right, while signal intensity decreased in the mPFC and dlPFC on the left (Figure 5). We also saw an increase in signal intensity in the left DMH, which fits with the role of this nucleus in the generation of autonomic responses to stress (DiMicco et al., 2002; Fontes et al., 2017). A brainstem-specific analysis also showed differential responses, with increases in BOLD signal intensity in RVLM and dlPons, as well as NTS (not shown), in the group exhibiting an increase in MSNA, while activity in the midbrain PAG only showed a sustained increase in the group in whom MSNA fell (Figure 6).

**FIGURE 7:**
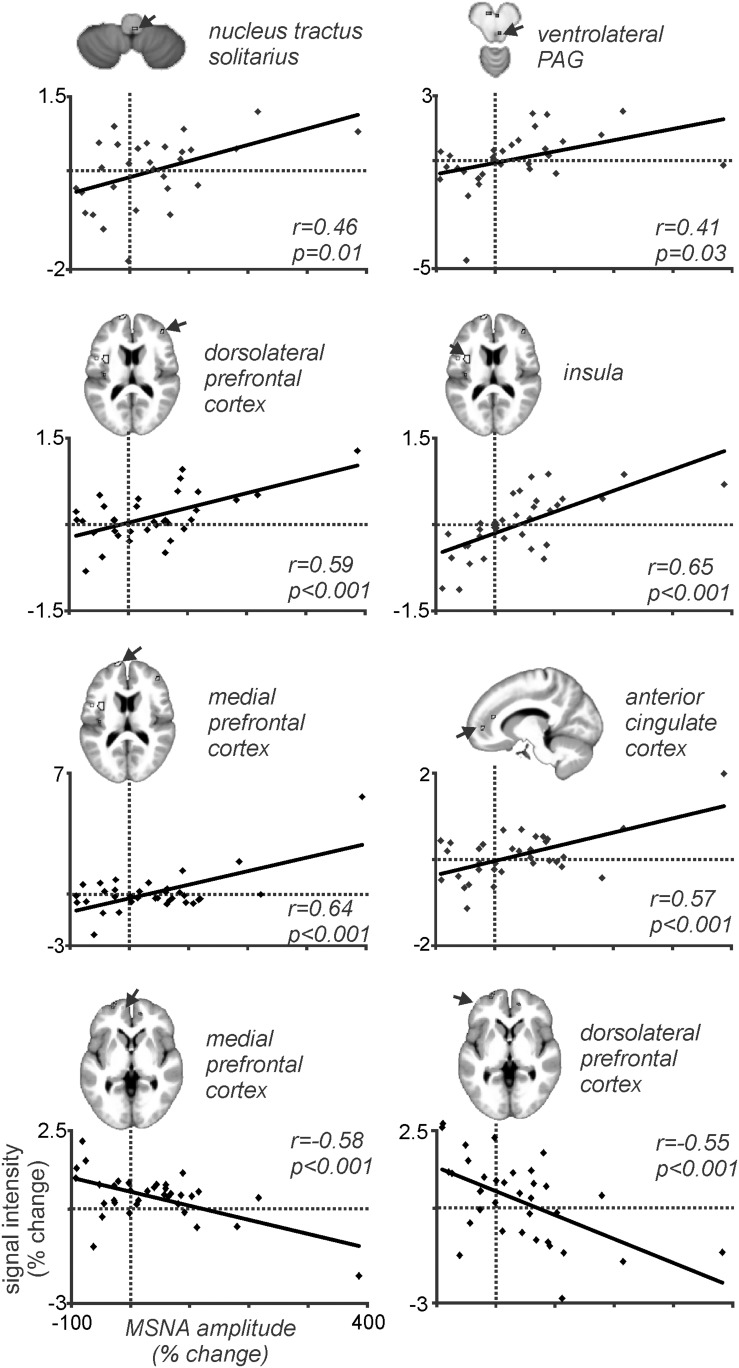
Plots of MSNA-coupled BOLD signal intensity during tonic pain in all 37 participants. In all regions there is a significant linear relationship between the change in BOLD signal intensity and the change in burst amplitude of MSNA. Reproduced, with permission, from Kobuch et al. (2018).

Because MSNA was recorded at the same time as we scanned the brain we could then correlate BOLD signal intensity to the pain-evoked changes in amplitude of MSNA. At rest, BOLD signal intensity was strongly coupled to bursts of MSNA in the RVLM, insula, dlPFC, PCC, and precuneus, and decreased in the region of the midbrain PAG. During pain, MSNA-coupled BOLD signal intensity was significantly higher in the region of the NTS and ventrolateral PAG on the right, dlPFC and ACC on the right, and insula and mPFC on the left; conversely, MSNA-coupled signal intensity decreased during pain in parts of the left dlPFC and mPFC (Kobuch et al., 2018). Mean data, showing the correlations between the change in BOLD signal intensity and change in MSNA burst amplitude, from 37 participants are illustrated in Figure 7. These results indicate that several areas of the brain are engaged in a burst-to-burst manner, with the magnitudes of these changes in signal intensity being correlated to the overall change in MSNA amplitude during tonic muscle pain (Kobuch et al., 2018). Interestingly, some important brain regions did not display pain-related changes. For example, while we had found that the RVLM and precuneus displayed strong coupling to MSNA at rest, during tonic muscle pain neither of these regions showed changes in signal intensity as a function of MSNA burst intensity. However, as noted above both of these regions displayed sustained signal intensity increases in the increasing MSNA group, and decreases in the decreasing MSNA group, suggesting that both RVLM and precuneus may provide a tonic modulatory role rather than changing in a burst-to-burst fashion during muscle pain (Kobuch et al., 2018).

## Changes in MSNA-Coupled Bold Signal Intensity During Pathophysiological Increases in MSNA

**FIGURE 8:**
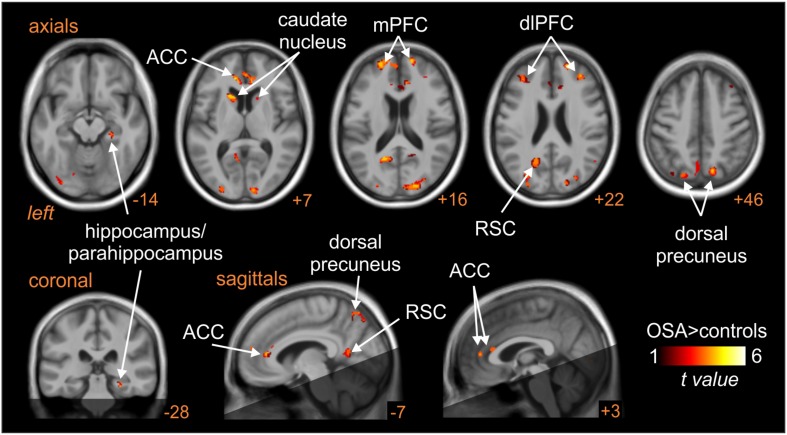
Brain regions in which MSNA-coupled changes in BOLD signal intensity were significantly different in OSA than controls. The dark shading indicates brain regions not included in the analysis. ACC, anterior cingulate cortex; dlPFC, dorsolateral prefrontal cortex; mPFC, medial prefrontal cortex; RSC, retrosplenial cortex. Reproduced, with permission, from Fatouleh et al. (2014).

Patients with obstructive sleep apnoea (OSA) have markedly elevated MSNA at rest, due to the repetitive episodes of nocturnal hypoxemia associated with collapse of the upper airways, which leads to neurogenic hypertension. This then is a pathophysiological model of high MSNA. As shown in Figure 8, MSNA-coupled BOLD signal intensity was higher in OSA than in controls in the following areas: dlPFC and mPFC bilaterally, dorsal precuneus, ACC, retrosplenial cortex (RSC), and caudate nucleus (Fatouleh et al., 2014). These data suggest that the elevated MSNA may be driven by changes in higher cortical regions, possibly through influences on brainstem nuclei.

**FIGURE 9:**
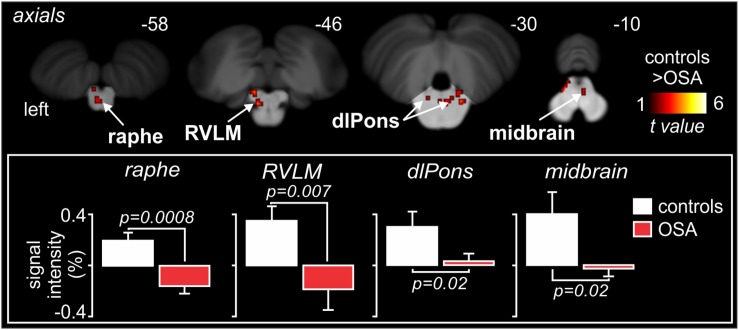
Brainstem regions in which MSNA-coupled BOLD signal intensity was significantly different in OSA and controls. Signal intensity increased in controls but decreased in OSA. Reproduced, with permission, from Lundblad et al. (2014).

Indeed, as shown in Figure 9, high-resolution scanning of the brainstem revealed significant coupling between spontaneous bursts of MSNA and BOLD signal intensity in a number of brainstem regions, including the medullary raphé, RVLM, dlPons, and midbrain, and significant increases in gray matter volume in the same areas (Lundblad et al., 2014). Although several mechanisms may lead to this increase in gray matter volume, it is possible that these changes are brought about by astrocytic activation and modulation of synaptic activity through altered gliotransmission. Indeed, it has been reported that chronic intermittent hypoxia is associated with activation of astrocytes in cortical regions such as the hippocampus (Aviles-Reyes et al., 2010). It is possible that repeated hypoxic events somehow evoke astrocyte activation in the raphe, dlPons and the RVLM which is consistent with increased gray matter density. Curiously, despite this increase in gray matter volume, MSNA-coupled BOLD signal intensity was actually *lower* in OSA than in controls, as shown in Figure 9. It is possible that reduced tonic inhibitory drive on rostral ventrolateral medullary premotor sympathetic neurones by the dlPons and medullary raphe leads to the increase in resting MSNA in OSA. Given that the BOLD signal is believed to reflect synaptic energy-dependent processes (Logothetis et al., 2001), a reduction in signal intensity within RVLM, despite an increase in output from this nucleus (MSNA was higher), may reflect a *reduction in active inhibition* onto the RVLM. Activation of astrocytes might then alter synaptic dynamics through the release of gliotransmitters such as glutamate, ATP or even GABA (Halassa et al., 2007; Ben Achour and Pascual, 2012). Regardless of the underlying mechanisms, our data show that there are changes in the brain that may be responsible for the increase in MSNA and blood pressure in OSA. In other words, *pathophysiological changes within the brain* lead to one of the clinical features of OSA – the hypertension. If this was true, we might expect that treatment of the condition would reverse these changes. Indeed, we showed that 6 months of continuous positive airway pressure (CPAP), which produced a significant fall in MSNA, caused reversal of the functional changes seen in OSA (Fatouleh et al., 2015; Lundblad et al., 2015).

## Conclusion

Muscle sympathetic nerve activity-coupled fMRI has allowed us to functionally identify the human RVLM and shown that its ongoing activity is coupled to several cortical and subcortical structures at rest. Moreover, the strength of this coupling can be modified by physiological or pathophysiological processes that lead to increases in MSNA. While physiological increases in MSNA may result in an increase in BOLD signal intensity of the RVLM, in the pathophysiological increase in MSNA seen in OSA it would appear that BOLD signal goes down, which we interpret as being due to a reduction in ongoing inhibition. Indeed, we suggest that the output of the human RVLM at rest is held in check by active inhibition, withdrawal of which can lead to increases in MSNA and blood pressure. Of course, we cannot exclude the possibility that other brainstem or hypothalamic areas contribute to the physiological or pathophysiological increases in MSNA, but the fact that we are seeing significant changes in RVLM, which receives inputs from many other brainstem and hypothalamic areas, leads us to conclude that much of what we see is indeed due to changes within RVLM.

## Author Contributions

This manuscript is a review of the authors’ collaborative work on the technique of MSNA-coupled fMRI. VM wrote the draft review, with contributions by LH.

## Conflict of Interest

The authors declare that the research was conducted in the absence of any commercial or financial relationships that could be construed as a potential conflict of interest.
